# Long-term esophageal and respiratory outcomes in children with esophageal atresia and tracheoesophageal fistula

**DOI:** 10.1093/gastro/gov055

**Published:** 2015-10-16

**Authors:** Richard H. Cartabuke, Rocio Lopez, Prashanthi N. Thota

**Affiliations:** ^1^Department of Internal Medicine, Cleveland Clinic, Cleveland, OH, USA,; ^2^Department of Biostatistics, Cleveland Clinic, Cleveland, OH, USA and; ^3^Department of Gastroenterology and Hepatology, Cleveland Clinic, Cleveland, OH, USA

**Keywords:** esophageal atresia, tracheoesophageal fistula, gastroesophageal reflux disease (GERD), esophageal dysmotility, aspiration pneumonia

## Abstract

**Objectives**: Few studies have evaluated the long-term complications and outcomes of esophageal atresia with or without tracheoesophageal fistula (EA/TEF) beyond childhood. The aim of our study was to characterize the esophageal and respiratory morbidity of EA/TEF through evaluation of clinical symptoms, diagnostic testing and therapeutic intervention at a tertiary care center.

**Methods**: Patients with congenital EA/TEF evaluated from 2011 to 2014 were included. Demographic characteristics, type and mode of repair of EA/TEF, clinical symptoms, radiographic, endoscopic, bronchoscopic and medication use data were obtained.

**Results**: A total of 43 patients were identified. The median age of this predominantly Caucasian population was 8 years (interquartile range: 3, 20). Twenty (62.5%) had type C (EA with distal TEF) abnormality. Twenty-one (48.8%) patients had heartburn, 19 (44.1%) had acid regurgitation, and 31 (72.1%) had dysphagia to solids. Barium swallow in 26 patients revealed strictures in 17 (65.4%), dysmotility in 20 (76.9%) and recurrent fistulas in four patients (15.4%). Thirty patients underwent upper endoscopy, of which 21 (70.0%) had a stricture, and six (20.0%) had recurrent fistula requiring surgical intervention. Eight (18.6%) patients underwent fundoplication. Pulmonary evaluation showed cough and choking in 31 (72.1%) patients and dyspnea and wheezing in 32 (53.4%) patients. Recurrent respiratory infections were reported in 19 (44.2%).patients. Other findings included tracheomalacia in 86.7% and restrictive lung disease in 54.5% of patients.

**Conclusion**: There is a high burden of residual esophageal and pulmonary pathology in patients with EA/TEF. Ongoing follow-up is required to monitor both the clinical symptoms and treatment responses.

## Summary Box Text:

### What is known

○ A majority of patients with esophageal atresia (EA) and congenital tracheoesophageal fistula (TEF) have chronic esophageal and respiratory symptoms despite surgical repair and thus require ongoing medical therapy.
○ Few studies have addressed the long-term outcomes of EA and/or TEF in a North American population.
○ Future studies are needed to address if early intervention to treat GERD will prevent long-term pulmonary damage.

### What is new

○ Esophageal and pulmonary morbidity persists in children and adolescents after surgical repair of EA and TEF.
○ A higher number of patients underwent fundoplication in our study compared with previously reported rates.
○ The findings in our North American study population are otherwise similar to the studies reported from European centers.

## Introduction

Esophageal atresia (EA) is a relatively common congenital malformation affecting approximately one in 2500 live births [1]. EA can occur with or without tracheoesophageal fistula (TEF). Most infants with EA/TEF require surgical repair in the first few days of life. The repair involves division and ligation of the TEF and primary esophageal anastomosis or lengthening procedures to complete anastomosis in patients with EA and wide separation between the proximal and distal esophageal segments [2].

Few studies have evaluated the long-term complications and outcomes of EA beyond childhood, especially as survivors of EA are reaching adulthood in increasing numbers [3]. Gastroesophageal problems including gastroesophageal reflux disease (GERD), esophageal strictures and esophageal dysmotility occur frequently following EA repair. Recent studies on long-term outcomes of TEF/EA patients have demonstrated esophagitis and intestinal metaplasia in a significant proportion [3].

In addition, respiratory sequelae including repeated infections, aspiration and persistent TEF may result in chronic pulmonary disease in survivors with EA and TEF. Structural anomalies persist in both the trachea and bronchus after surgical repair. Tracheomalacia is common because of the longer and more compliant membranous portion of the posterior wall of the trachea and can lead to poor secretion clearance and recurrent pneumonia [4]. Abnormal swallowing, GERD and the recurrence of a TEF at the previous surgical repair site can all lead to aspiration and recurrent lower respiratory tract infection (LRTI).

Most of the published literature is from Europe, with very few studies addressing the long-term outcomes in a North American population. Therefore, our study aims were: (i) to evaluate esophageal and pulmonary symptoms in patients with EA/TEF at our institution, and (ii) to review the diagnostic testing and therapeutic interventions in this population.

## Methods

After approval was obtained from the Institutional Review Board (IRB) at Cleveland Clinic, patients with the diagnosis of esophageal atresia and/or congenital tracheoesophageal fistula were identified using the Cleveland Clinic eResearch system for the period beginning 1 January 2011 and ending 1 October 2014. Chart review was performed to confirm diagnosis before patients were included in the study.

The following data were obtained: (i) demographic characteristics (age, race and sex); (ii) type of EA and/or TEF and mode of repair including the primary repair, elongation of the esophagus for long-gap, interposition of the colon and gastric transposition; (iii) esophageal symptoms (heartburn, acid regurgitation, dysphagia to solids or liquids or chest pain); (iv) pulmonary symptoms (shortness of breath, wheezing, cough, choking, diagnosis of asthma and recurrent respiratory infections); (v) use of medications (proton pump inhibitors, H2 blockers, inhaled bronchodilators, inhaled corticosteroids and systemic steroids); (vi) diagnostic evaluations (barium swallow, esophageal manometry, gastric emptying study and upper endoscopy along with any therapeutic interventions); and (vii) pulmonary testing (computed tomography [CT] of the chest, pulmonary function testing with or without lung diffusion capacity and bronchoscopy with or without bronchoalveolar lavage).

A clinical diagnosis of GERD was made based on the presence of typical symptoms of heartburn or acid regurgitation. Patients were diagnosed with asthma if they had symptoms typical of asthma with the finding of wheezing on physical examination. Follow-up time was defined as time since birth to event; subjects were censored at death or 1 October 2014.

## Statistical analysis

Data are presented as mean ± standard deviation, median (25th and 75th percentiles) or number (%). Time-to-event analysis was performed to assess occurrence of primary repair, elongation of esophagus, fundoplication, abnormal barium swallow, abnormal manometry and survival. All analyses were performed using SAS (version 9.4, The SAS Institute, Cary, NC).

## Results

### Demographic characteristics

A total of 43 patients with the diagnosis of congenital EA and/or TEF were seen at our institution during the study period (Table 1). The median age at the end of follow-up was 8 years (interquartile range: 3–20). The patients were predominantly Caucasian. An equal sex distribution was noted with a male-to-female ratio of 21:22.

**Table 1.** Demographic characteristics and tracheoesophageal atresia/fistula repair data

Factor	Number	Summary
Age (years)	43	8 (3, 20)
Race	43	
Black or African American		3 (7.0)
White		37 (86.0)
More than one race		1 (2.3)
Hispanic or Latino		2 (4.7)
Sex	43	
Female		22 (51.2)
Male		21 (48.8)
Type of EA	32	
Type A: EA without TEF		7 (21.9)
Type B: EA with proximal TEF		4 (12.5)
Type C: EA with distal TEF		20 (62.5)
Type E: TEF with EA (H-Type)		1 (3.1)
Age at repair (days)	39	
0–7 days old		28 (71.8)
8–14 days old		2 (5.1)
15–60 days old		3 (7.7)
61–180 days old		2 (5.1)
>180 days old		4 (10.3)

Values presented as median (interquartile range) or number (column %).

EA: esophageal atresia. TEF: tracheoesophageal fistula

### Type of tracheoesophageal atresia/fistula and repair characteristics

Data pertaining to the type of esophageal atresia and/or tracheoesophageal fistula were available for 32 patients (Table 1). The most prevalent type of TEF was type C, which was seen in 20 (62.5%) patients. Types A, B and E were less commonly observed. Information regarding type of surgical repair was available for 39 patients. A majority of patients (71.8%) underwent repair within the first week of life. Thirty-three patients underwent primary repair, with six requiring subsequent esophageal lengthening surgery for a long gap. Five patients underwent interposition of the colon, and one had a gastric transposition. Seven patients required tracheostomy for breathing difficulties.

### Esophageal evaluation

A majority of patients had symptoms of heartburn, acid regurgitation and dysphagia to both solids and liquids (Table 2). Chest pain was described in eight patients. Esophageal stricture was a common finding on barium swallow (65.4%). Three patients had esophageal manometry, with two demonstrating absent peristalsis and one demonstrating ineffective esophageal motility. Of the nine patients who underwent a gastric emptying study, four demonstrated delayed gastric emptying. Of the 30 patients who had an upper endoscopy, 21 (70.0%) were found to have a stricture requiring dilation. Six (20.0%) patients had recurrent tracheoesophageal fistula and underwent further surgical intervention. Two (6.7%) patients were found to have Barrett’s esophagus with no dysplasia noted in either case.

**Table 2.** Esophageal clinical, radiologic and endoscopic data

Factor	Number	Summary, n (%)
Clinical features	43	
Heartburn		21 (48.8)
Acid regurgitation		19 (44.2)
Dysphagia to solids		31 (72.1)
Dysphagia to liquids		19 (44.2)
Chest pain		8 (18.6)
Barium swallow findings	26	
Stricture		17 (65.4)
Recurrent fistula		4 (15.4)
Dysmotility		20 (76.9)
Esophageal manometry testing	3	
Absent peristalsis		2 (66.7)
Weak peristalsis		1 (33.3)
Esophagogastroduodenoscopy findings	30	
Stricture		21 (70.0)
Recurrent fistula		6 (20.0)
Barrett’s esophagus		2 (6.7)

Over the follow-up period, eight (18.6%) patients had fundoplication. All of the eight patients had fundoplication elsewhere prior to receiving clinical care at our facility, with limited access to data for pre-operative esophageal pH monitoring and manometry performed at other institutions. Seven patients had a complete Nissen fundoplication, and one had a partial fundoplication. Ever-use of proton-pump inhibitors (PPIs) was reported in 33 patients and ever-use of H_2_ blockers in 26 patients.

### Respiratory evaluation

The most common pulmonary symptoms were shortness of breath, wheezing, cough and choking (Table 3). Recurrent respiratory infections were seen in up to 44.2% of patients. Eleven (25.6%) patients had the diagnosis of asthma. Often, these young patients were unable to tolerate spirometry and were thus treated symptomatically.

**Table 3.** Respiratory evaluation data

Factor	Number	Summary, n (%)
Clinical features	43	
Shortness of breath		23 (53.5)
Wheezing		23 (53.5)
Cough		31 (72.1)
Choking		31 (72.1)
Diagnosis of asthma		11 (25.6)
Recurrent respiratory infection		19 (44.2)
Chest CT	15	
Aspiration pneumonia		5 (33.3)
Tracheomalacia		7 (46.7)
Bronchomalacia		1 (6.7)
Bronchiectasis		4 (26.7)
Bronchitis		1 (6.7)
Pulmonary function testing	11	
Restrictive		6 (54.5)
Mixed		3 (27.3)
Normal		2 (18.2)
Bronchoscopy	15	
Tracheomalacia		13 (86.7)
Tracheal stenosis		4 (26.7)
Recurrent tracheoesophageal fistula		4 (26.7)
Bronchomalacia		2 (13.3)

During follow-up, 15 patients underwent chest CT. The findings included pneumonia (likely from aspiration) in five patients, tracheomalacia in seven patients, bronchiectasis in four patients, bronchomalacia in one patient and bronchitis in one patient. Pulmonary function testing was available for 11 patients, with a majority of patients (54.5%) demonstrating a restrictive pattern. Lung diffusion capacity was significantly decreased in seven patients. Fifteen patients underwent bronchoscopy with 13 (86.7%) demonstrating tracheomalacia, four with evidence of tracheal stenosis, four with evidence of recurrent TEF and two demonstrating bronchomalacia. Twenty-nine patients had prior use of inhaled bronchodilators. Twenty patients had previously used inhaled corticosteroids, and 20 used systemic steroid therapy.

There were seven patients in our study with EA without TEF (type A), among which four had pulmonary function testing at our institution. One patient had a restrictive pattern, one had a mixed pattern, and two were normal. Recurrent pulmonary infections were seen in five out of seven patients with EA without TEF (type A). In comparison, all patients with EA and TEF had abnormal pulmonary function testing. Fourteen out of 36 patients with TEF had recurrent respiratory infections. All six patients with recurrent TEF had multiple respiratory infections. These findings suggest that the presence of TEF was associated with higher pulmonary morbidity.

### Mortality

Three patients died during the follow-up period. The first patient died two weeks postoperatively after primary surgical repair for TEF from overwhelming sepsis, which was secondary to known gastrointestinal perforation confirmed by abdominal imaging. The second patient died of respiratory compromise secondary to aspiration pneumonia prior to TEF repair. The third patient suffered cardiac arrest after prolonged intubation.

## Discussion

This study examined the frequency of clinical symptoms and pharmacotherapy as well as the frequency of abnormal findings in a battery of esophageal and pulmonary tests in children and adolescents after surgical repair of EA and TEF. Long-term follow-up of patients with EA and/or TEF revealed that over 80% had dysphagia and more than 50% had GERD. Esophageal stricture and dysmotility were common findings on barium swallow. A majority of patients were maintained on medical therapy for GERD, with 19% requiring fundoplication during the follow-up period. More than half of the patients in our study cohort had chronic cough, choking episodes, shortness of breath, wheezing and recurrent respiratory infections. Approximately 25% of patients had the diagnosis of asthma. Tracheomalacia was a common finding on chest CT and bronchoscopy. The majority of patients who had pulmonary function testing performed revealed a restrictive pattern of disease.

Our study parallels the findings suggested by other studies regarding the long-term esophageal and pulmonary complications of congenital EA/TEF. EA/TEF is associated with a multitude of esophageal symptoms including: heartburn, acid regurgitation, dysphagia to solids and or liquids and chest pain. The incidence of GERD ranges up to 63% in some studies, with an even higher number of patients (up to 77%) with complaints of dysphagia [5–12]. Dysmotility is also a common finding in patients who have undergone primary EA/TEF repair [3]. While esophageal manometry was not commonly performed in our study, the patients undergoing such testing demonstrated ineffective or absent peristalsis. In our study, 18.6% of patients underwent fundoplication during the follow-up period, which is more than that reported in previous literature. Additionally, underlying GERD is often associated with strictures requiring dilation, which was needed in 49% (21/43) of our patient population (with many individuals requiring additional procedures). As such, there may be a role for early fundoplication surgery in patients who fail medical therapy [13].

The relationship between chronic GERD and aspiration has been well established. The majority of patients demonstrate restrictive physiology on pulmonary function testing, suggesting a causal link between chronic GERD and dysmotility as it relates to aspiration. Notably, one previous study by Peetsold *et al.* found that a restrictive pattern on pulmonary function testing was present in both groups of patients who had or had not undergone anti-reflux surgery for GERD, but the former group had significantly lower FEV1 (forced expiratory volume in one second) values [4]. Studies investigating fundoplication in patients with GERD and esophageal dysmotility are needed. Interestingly, a majority of patients used inhaled bronchodilators and corticosteroids, but it remains unclear if their use correlated with a change in the frequency of GERD and/or aspiration symptoms such as cough or choking. Data reporting perioperative complications are scarce; however, recurrent TEF after primary repair was noted in 3%–4% of patients in previous studies [14,15]. This is in stark contrast to our study, which demonstrated recurrent TEF in 14% (6/43) of patients. As a large academic referral center, the patients with recurrent TEF were transferred to our facility for further management, which may explain the higher incidence of recurrent TEF noted in our study.

The respiratory complications in children and adolescents with EA and/or TEF are well documented and included shortness of breath, wheezing, cough, choking, diagnosis of asthma and recurrent respiratory infections. Our study demonstrated a higher proportion of patients with cough/choking and shortness of breath than has been reported previously. The prevalence of asthma after repair of EA and/or TEF has ranged from 12%–29% [16–18]. In our study, 25.6% of patients carried the diagnosed of asthma, and almost all were being treated with inhaled bronchodilators. Twelve patients had pulmonary function testing during follow-up with 55% demonstrating a restrictive pattern, 27% demonstrating a mixed pattern and 18% with a normal study, which is similar to the incidence reported in other studies. This may be related to permanent lung damage caused by chronic aspiration with superimposed respiratory infections. The role of bronchoscopy may guide further investigation, especially in patients with tracheomalacia seen on computed tomography, as these patients tend to have more respiratory complaints [4].

There are sparse data on the outcomes of children and adolescents after surgical repair of EA and TEF in the North American population. Our study addresses this issue. As both esophageal and tracheal anomalies are interrelated, we sought to study long-term sequelae on upper digestive and respiratory systems. Currently, there are no published guidelines regarding surveillance for patients with EA and/or TEF. We recommend endoscopic evaluation for patients presenting with esophageal symptoms as sequelae (including esophageal strictures and recurrent TEF) are common. Additionally, considering the high prevalence of impaired lung function seen on pulmonary function tests within this patient population, yearly pulmonary function tests would be advisable for early detection and monitoring. In patients with continued GERD in spite of aggressive medical management, fundoplication is advised after preoperative testing with esophageal manometry and esophageal pH testing.

The limitations of the study are those related to the retrospective nature of the data collection with missing data regarding the details of the initial repair and perioperative complications. There may also be referral bias, as the patients seen in our tertiary referral center may not be representative of the total population.

There is a high burden of residual esophageal and pulmonary pathology in patients with EA/TEF requiring medical and surgical interventions. Continual monitoring of clinical symptoms and treatment response is required. It is not clear if early aggressive therapy for GERD may prevent the pulmonary and esophageal complications as they relate to EA and/or TEF. Further studies are required to evaluate the role of early fundoplication for preventing future pulmonary morbidity.
